# Obituary

**Published:** 1888-12

**Authors:** 


### OBITUARY.

### THE LATE DR. GEORGE W. KEELY

The death of Dr. Keely, occurring as it did just before the convention of the American Dental Association, cast a gloom over the opening exercises of that body. Dr. Keely had long been a member, and at one time its honored president. At the time of his death he was its treasurer, a position which he had held for a number of years. The sad accident that led to his death was a fall from the third-story window of his dwelling, while he was trying to mend a telephone wire connected with the office. In addition to internal injuries received by him in this fall of over thirty feet, by a singular accident an open penknife, which he held in his hand, penetrated the base of the skull, entering over half an inch into the brain. The knife was broken off in his endeavors to remove it, and it finally had to be chiselled out, so firmly was it imbedded in the skull. The accident occurred on Wednesday evening, August 22, he, however, retained consciousness up to a short time before his death, passing quietly away at 2 P. M. on the Friday following.

Dr. Keely was born at Oxford, Ohio, Oct. 22, 1822. In 1839 he entered the dental office of Dr. John Allen, of Cincinnati, with whom he spent two years. Returning to Oxford in 1841 he started a dental office at that place. After having been in active practice for about twelve years, in 1853 Dr. Keely graduated at the Ohio College of Dental Surgery. He was present at the first meeting of dentists held at Niagara Falls in 1859, where the foundation was laid for the organization of the American Dental Association. He was an active or honory member of the Mississippi Valley, Mad River Valley, Indiana, Kentucky, Illinois, Missouri and Wisconsin State Dental Societies, and of the New York Odontological Society. He was one of the trustees of The Ohio College of Dental Surgery, and lecturer on Irregularities of the Teeth in the same institution. He was married in 1851 to Miss Susanna Wells, of Cincinnati, who died in 1856. Dr. Keely was again married in 1861 to Miss Cornelia Cone, of Oxford, O. Of the three children by his first wife, but one, Dr. Chas. I. Keely, of Hamilton, survives him, and of the eight by his second wife, but three remain to mourn his loss.

Of his personal character, Dr. Geo. Watt, who knew him intimately, thus feelingly speaks: "Our acquaintance with Dr. Keely began in 1853, and was intimate from that date forward. He was a true and trusted friend, and we think we have reason to know the manner of man he was, the strength of his friendship, the generosity of his nature, the integrity of his purposes and the deep sincerity of his life. He was a man of solid attainments; a man of rare character and genuine worth; a recognized master in his specialty of dentistry, and yet characterized by that modesty and self-forgetfulness and simplicity that so often accompanies true greatness. He was an enthusiast in his calling, and saved neither toil, time nor expense in keeping abreast with the progress of the profession. He never had a secret that would be helpful to others, that he did not labor industriously to communicate to his professional brethren. Of the extent and character of his writings, the readers of the *Ohio Journal* are enabled to judge from what have appeared in its columns during the eight years of its existence."

The following resolutions were offered and adopted by the American Dental Association, at Louisville, Aug. 30th, 1888.

WHEREAS, It having pleased Almighty God in whose hand is the breath of every living thing, to remove suddenly from the scene of his toils and his honors, our friend and associate, Geo. W. Keely, D.D.S.; therefore be it

*Resolved*, As the sense of this Association, that in Dr. Keely's death this Association has lost one of its oldest and most valued members, a former president, and for many years its treasurer. Genial and urbane in disposition, kind and affectionate in his family, eminently successful in his calling, Dr. Keely was one whom we loved as a man and honored as a dentist. Ourselves marching to the eternal world, let us loiter for a moment on the busy highway of life to hang this garland on his tombstone.

*Resolved*, That these resolutions be engrossed upon the records of this Association and a copy be forwarded to his family.

H. A. Smith, Cincinnati,<br>

E. T. Darby, Philadelphia, } Committee.<br>

G. J. Friedrichs, New Orleans,

